# Effect of sprouted whole pearl millet on growth performance, intestinal development, bacterial count, and blood indices of broiler chickens

**DOI:** 10.1093/tas/txad045

**Published:** 2023-05-08

**Authors:** Oluwakemi Olasehinde, Foluke Aderemi

**Affiliations:** Department of Animal Science, College of Agriculture, Engineering and Science, Bowen University, Iwo, Nigeria; Department of Animal Science, College of Agriculture, Engineering and Science, Bowen University, Iwo, Nigeria

**Keywords:** broiler chicken, blood indices, ceca bacterial, intestinal morphology, intestinal pH, sprouted pearl millet

## Abstract

This study investigated the effects of varying levels of sprouted whole grain pearl millet (SPM) on growth performance, intestinal morphology, microbial count, and blood indices of broiler chickens. A maize–soybean meal basal diet was formulated and fed to broiler chickens as starter (0 to 21 d) and finisher (22 to 42 d) diets. The diets comprised of 0%, 25%, 50%, 75%, and 100% of SPM incorporated as whole grain. On 0 d, 180 unsexed broiler chickens were allocated to experimental diets in a completely randomized design. Each treatment was replicated three times; each replicate had 12 chicks. All diets were isonitrogenous and isocaloric to meet the nutrient requirements of broiler chickens. Diets and water were provided ad libitum for 42 d.

Results showed that the body weight gain (BWG) of broiler chickens on SPM compared favorably with those on the control diet. BWG showed trends in increment (*P* < 0.10) while FCR showed decreased trends (*P* < 0.10) with partial inclusion of SPM at 42 d and 0 to 42 d. The drumstick weight showed quadratic effect (*P* = 0.044) while the wing weight showed linear effect (*P* = 0.047) to treatment diets at 21 d. The liver weights of broiler chickens showed linear response (*P* = 0.018) at 21 d and (*P* = 0.004) at 42 d to SPM inclusion in diets. Sprouted whole PM consistently increased low-density lipoprotein concentration and mean corpuscular hemoglobin concentration (*P* < 0.05). Length and weight of small intestine and ceca showed decreasing trends on SPM levels in the treatment diets. Digesta pH assessment revealed that pH in the crop was lower (*P* < 0.05) on partial SPM while pH in proventriculus was reduced (*P* < 0.05) with inclusion of SPM in treatment diets. Lactobacilli count decreased linearly (*P* = 0.010) with SPM inclusion. This study suggests that SPM could be used as an alternative source of energy in production of broiler chickens. Therefore, partial replacement of maize with SPM in broiler diet had no negative effect on performance, physiological status, and overall health of broiler chickens.

## INTRODUCTION

Rapid increase in the price of feed ingredients and demand for food raises the question of how poultry production can meet the global demand for protein. Chicken meat is widely consumed and is currently the largest produced meat globally (Shahbandeh, 2022). Among poultry products supplying protein to human population, broiler meat represents a great source of inexpensive animal protein (Elahi et al., 2020), which plays an important role to meet growing global demand for poultry products.

Maize grain remains the main energy source in commercial poultry broiler diets, a major challenge for poultry producers. Due to increased demand for maize grain as a staple food for both animals and humans in sub-Saharan Africa, high cost of production, adverse climate and growth conditions, and demand by industries for biofuel have resulted in high market prices. It is therefore crucial that alternative dietary energy sources be identified as possible replacement for maize (Khan, 2018; Parolini et al., 2020).

Pearl millet (*Pennisetum glaucum*, PM) is a drought-tolerant crop and constitutes a major source of carbohydrates and proteins in the semiarid tropics of Africa and Asia. Pearl millet is proposed to replace maize grain in poultry diets because of its comparable energy and nutrient profile. It is also rich in nutrients, fiber, and natural antioxidant compounds (Punia et al., 2021). Despite PM as dietary energy source that could replace maize in broiler diets (Baurhoo et al., 2011; Cisse et al., 2017), the results of studies where maize grain was replaced with PM grain have been inconsistent (Baurhoo et al., 2011; Cisse et al., 2017; Adeleye et al., 2020).

Cereals contain compounds referred to as antinutritional factors (ANFs) that affect feed intake, metabolism, and availability of nutrients in animals. The presence of ANFs in PM including phytate, tannins, and polyphenols (Boncompagni et al., 2018; Rani, 2018), results in chelation of dietary minerals in the gastrointestinal tract, thereby reducing their bioavailability (Nour et al., 2014). Processing methods such as sprouting, fermentation, mechanical, and thermal treatments of PM grains improve nutritional quality, digestibility, bioavailability of nutrient, and reduce ANFs (Khempaka et al., 2014; Sugiharto et al., 2016). Sprouting has been shown to improve nutritional value of grains by converting complex compounds into simpler forms thereby improving their digestibility and utilization (Oghbaei and Prakash, 2016). In addition, sprouting can increase PM grain nutrient quality and improve nutrient utilization (Preetika et al., 2004; Younis et al., 2019). However, limited research has been conducted to determine the effect of processing PM grain through sprouting on the growth performance, productivity, and blood traits of broiler chickens. Therefore, this study investigated the effect of replacement of maize grain with different levels of sprouted PM grains on growth performance, intestinal development, bacterial count, and blood indices of broiler chickens.

## MATERIALS AND METHODS

### Ethical Approval, Animal Study and Location of the Study Area

The animal routine care, use, and its experimental protocols were approved by the institutional research and ethics committee (Bowen University, Iwo, Osun State, Nigeria). It was performed in accordance with the standard guidelines of the University and conformed to ARRIVE 2.0 and NRC guidelines (NRC, 2011; Du Sert et al., 2020) and measures were taken to reduce pain or discomfort in the broiler chickens. The study was carried out at the Teaching and Research Farm of the Department of Animal Science, Bowen University, Iwo, Nigeria.

### Experimental Animals, Management, and Design

One hundred and eighty (*n* = 180) broiler chickens were procured from commercial hatchery (Amo Farm Sieberer Hatchery Limited, Along Ife Odan Road, Oyo, Nigeria) and transported on day of hatch to Bowen University Teaching and Research farm. Upon arrival, the broiler chickens were tagged, weighed, and allocated randomly into 5 treatment groups with 3 replicates of 12 broiler chickens each in a completely randomized design. The broiler chickens were raised in cages with wire meshed floor measuring 90 cm× 50 cm× 50 cm. The experimental diets and water were offered ad libitum throughout the study period. Broiler chickens in all replicates were reared under the same environmental and management conditions. The broiler chickens were subjected to two lighting programs 23L: 1D (0 to 7 d after hatch) and 20L: 4D (8 to 42 d, experimental period). Temperature during experimental period ranged between 24 and 37 °C, while the humidity was between 25% and 92%. Electric fans were provided to reduce the environmental temperature within the pen. Broiler chickens were monitored for their health twice daily. The broiler chickens were randomly assigned to dietary treatment based on their body weights determined according to the ARRIVE 2.0 guidelines and Experimental Animal Allotment Program (Kim & Lindermann, 2007; Du Sert et al., 2020). The number of animals per group was calculated based on the research of Baurhoo et al. (2011). Data on growth performance had a mean difference of 453.84 between control and experimental diets and standard error of mean of 96.70. Sample size selected was six broiler chickens per group based on 80% confidence interval and *P*-value of 0.05.

### Dietary Treatments

The PM grains were purchased from local market in Lagos, Nigeria. The grains were cleaned of all debris and surface sterilized in saline solution with shaking every 15 min. The brine solution was drained after 30 min, and sterilized grains were rinsed with distilled water three times before sprouting. The grains were left to sprout on jute bag at room temperature in the dark for 3 d following the method previously described (Afsharmanesh et al., 2016). Temperature during sprouting period ranged between 27 and 33 °C. The PM sprouts were between 1 and 3 mm, with most of the sprouts at <2 mm long (Sibbald et al., 1962). The sprouts were sun-dried thoroughly for 2 d and stored in cool dry place until preparation of experimental diets.

The proximate composition of SPM is given in Table 1. The nutritional composition of formulated control and SPM diets is shown in Table 2. All diets were maize—–soybeans based and were provided in mash form. A two-phase feeding program with starter diet from 0 to 21 d and a finisher diet from 22 to 42 d was adopted in this study. Five experimental diets were formulated for the starter phase and the finisher phase, to meet or exceed NRC (1994) nutritional requirements for broiler chickens. The control diet contained 100% maize (0), diet 2 contained 25% whole SPM (25), diet 3 contained 50% whole SPM (50), diet 4 contained 75% whole SPM (75), and diet 5 contained 100% whole SPM (100).

**Table 1.** Chemical composition of sprouted whole pearl millet

Item	Sprouted PM
Metabolizable energy, kcal/kg	3,369.90
Protein, %	10.80
Dry matter, %	88.40
Ash, %	1.30
Crude fat, %	5.51
Crude fiber, %	1.90
Available phosphorus, %	0.10
Phytate phosphorus, %	0.22

PM, pearl millet.

**Table 2.** Ingredients and chemical composition of experimental starter (0 to 21 d) and finisher (22 to 42 d) diets

Item	Starter (0 to 21 d)					Finisher (22 to 42 d)				
SPM^1^, %					SPM^1^, %				

	0	25	50	75	100	0	25	50	75	100
Maize	52.07	39.60	26.61	15.62	-	59.80	45.49	30.92	17.28	-
Pearl millet	-	14.12	28.78	41.20	58.84	-	16.14	32.59	47.99	67.50
Soybean meal	40.91	39.66	38.40	37.33	35.81	34.00	32.61	31.19	29.88	28.18
Soybean oil	2.69	2.26	1.83	1.46	0.94	2.15	1.69	1.21	0.76	0.18
Salt	0.25	0.25	0.25	0.25	0.25	0.25	0.25	0.25	0.25	0.25
DCP^2^	2.16	2.15	2.13	2.11	2.09	1.95	1.93	1.91	1.89	1.86
Limestone	1.49	1.51	1.53	1.55	1.57	1.39	1.41	1.43	1.43	1.48
Vit-Min premix^3^	0.25	0.25	0.25	0.25	0.25	0.25	0.25	0.25	0.25	0.25
Lysine	—	0.03	0.05	0.07	0.10	0.05	0.08	0.11	0.13	0.17
Methionine	0.18	0. 17	0.17	0.16	0.15	0.16	0.15	0.14	0.14	0.13
Calculated composition										
ME^4^, kcal/kg	2,970	2,970	2,970	2,970	2,970	3,004	3,004	3,004	3,004	3,004
Crude protein	22.50	22.50	22.50	22.50	22.05	20.00	20.00	20.00	20.00	20.00
Methionine, %	0.50	0.50	0.50	0.50	0.50	0.45	0.45	0.45	0.45	0.45
Lysine, %	1.21	1.21	1.21	1.21	1.21	1.10	1.10	1.10	1.10	1.10
Calcium, %	1.10	1.10	1.10	1.10	1.10	1.00	1.00	1.00	1.00	1.00
Phosphorus, %	0.50	0.50	0.50	0.50	0.50	0.45	0.45	0.45	0.45	0.45

^1^SPM, sprouted pearl millet.

^2^DCP, dicalcium phosphate.

^3^vitamin–mineral premix supplied per kilogram of diet: vitamin A, 30,000 IU; vitamin D3, 6,250 IU; vitamin K, 5 mg; vitamin E, 75 mg; vitamin B1, 5.63 mg; vitamin B2, 15 ; vitamin B6, 11.25 mg; vitamin B12, 0.0375 mcg; niacin, 100 mg; pantothenic acid, 37.5 mg; folic acid, 3.75 mg; choline chloride, 750 mg; manganese, 200 mg; biotin, 0.125 mcg; zinc, 125 mg; iodine, 2.5 mg; copper, 12.5 ; selenium, 0.5 mg; cobalt, 1.25 mg; iron, 50 mg; antioxidant, 312.5 mg.

^4^ME, metabolizable energy.

### Growth Performance, Carcass, Organ, and Intestinal Measurements

Daily feed intake (FI) and body weight (BW) of the broiler chickens were recorded until 42 d. The resulting data were used to calculate body weight gain (BWG), FI, and feed conversion ratio (FCR) for each growth phase. FCR was obtained as the ratio of FI to BWG.

At both phases, broiler chickens (*n* = 30, 6 broiler chickens per treatment) were subjected to overnight fasting, weighed, and sacrificed to determine carcass, digestive, and immune organ weights. Cold defeathering was carried out on all broiler chickens. Carcasses were cut into different parts. The weight of breast, drumsticks, thigh, wings, and abdominal fats were recorded to determine corresponding relative weights. The weight of the empty proventriculus, empty gizzard, pancreas, liver, spleen, bursa of Fabricius, and thymus were measured on a digital scale (Kern, GmbH, Germany) with an accuracy of 0.01 g. The carcass and organ relative weights were calculated based on carcass and organ weight multiplied by 100 and divided by the BW of each bird.

The entire intestinal segments were collected and placed on a tray at room temperature, then gently uncoiled to avoid tearing or stretching, intestines (duodenum, jejunum, ileum, and ceca) were removed immediately after sacrifice and weighed individually in a digital scale (Kern, GmbH, Germany) of 0.01 g precision. The small intestine length was cut into sections of duodenum, jejunum, and ileum and measured with the aid of a cutting mat. The relative weight (g/g) and length (cm/g) of each section were then estimated.

### Blood Collection and Analysis

At 21 d, blood samples (*n* = 30, 6 broiler chickens per treatment) were drawn from wing veins into vacutainer serum tubes and whole blood was drawn into tubes containing anticoagulant (ethylene diamine tetraacetate acid). Serum samples were centrifuged at 3,000 × *g* at 4 °C for 5 min and stored at −20 °C until further analysis. Low-density lipoprotein (LDL), high-density lipoprotein (HDL), triglycerides, cholesterol, glucose, albumin, total protein, aspartate transaminase (AST), alanine transaminase (ALT), and alanine phosphatase were measured from serum. Whole blood samples were stored at 4 °C until analysis for hemoglobin (Hb), packed cell volume (PCV), red blood cell (RBC), white blood cell (WBC) and differentials, mean corpuscular volume (MCV), mean corpuscular hemoglobin (MCH), mean corpuscular hemoglobin concentration (MCHC), AST, and ALT. In order to determine serum and whole blood indices, a commercial kit (RANDOX Laboratory Ltd, UK and AGAPPE Diagnostics Switzerland GmbH) was used and analyses were carried out according to the manufacturer’s instructions based on previously described protocol. The MCV, MCH, and MCHC were determined from formulae established by Jain (1986). The data on level of globulin were obtained from serum total protein minus albumin.

### Intestinal pH

Sampling was carried out at 21 and 42 d at the same time each sampling day with 30 broiler chickens (*n* = 30, 6 broiler chickens per treatment) randomly selected for intestinal pH measurement. Immediately post euthanasia, the intestinal tract from the crop to ceca was excised intact. The fat and mesentery were separated from the intestinal segments. The pH of intestinal segments crop, proventriculus, gizzard, small intestine, and ceca of each broiler chicken was determined by inserting a digital pH electrode (Smart Spear pH tester, PH60S-Z Apera Instruments, USA) into a small opening made in each section. Care was taken to ensure pH electrode did not touch the wall of each section. Six readings were taken from each section after values stabilized on the screen of the pH meter. After reading from each segment the electrode was rinsed with distilled water and stored according to manufacturer instructions.

### Microbiological Analysis

At 21 d, 30 broiler chickens (*n* = 30, 6 broiler chickens per treatment) were randomly selected to determine *Salmonella* and *Lactobacilli* count in the ceca. Ceca contents were decanted into separate sterile plastic containers and then thoroughly mixed up. The ceca contents were subsequently analyzed. Ten grams of homogenized sample along with 10-fold serial dilutions were taken for enumeration of *Lactobacillu* and *Salmonella*. The microbiological analysis was performed in duplicate and mean values were incorporated for statistical analysis. *Lactobacilli* were determined using De Man, Rogosa, and Sharpe agar (Merck, Germany) and incubated at 37 °C for 48 h. *Salmonella* was enumerated using Bismuth Sulfate Agar at 37 °C for 24 h. The bacterial colony forming unit (CFU) were counted using colony counter and reported as log_10_CFU per 1 g of sample.

### Statistical Analyses

Data analyses were carried out with GenStat Statistical Software (GenStat 21st for Windows, Rothamstead, Herefordshire, UK) using a completely randomized design. The bacteriological data required log transformation before statistical analysis. Orthogonal polynomial (linear and quadratic) was used to examine the dose response due to increasing amounts of SPM in the diet. *P* ≤ 0.05 was considered to be statistically significant and *P* < 0.10 considered as trends.

## RESULTS

The growth performance of broiler chickens fed whole SPM-based diets are presented in Table 3. The inclusion of SPM in the diets of broiler chickens has no effect (*P* > 0.05) on BWG, FI, and FCR. However, BWG at 22 to 42 d (*P* = 0.072) and at 0 to 42 d (*P* < 0.055) showed increasing trend of improvement on SPM diets. Similarly, FCR at 22 to 42 d (*P* < 0.059) and at 0 to 42 d (*P* < 0.058) showed decreasing trends.

**Table 3.** Growth performance of broiler chickens fed sprouted whole PM-based diets

Item	SPM^1^, %					SEM^2^	*P*-value^3^	
	0	25	50	75	100		Linear	Quadratic
BWG^4^, g/bird								
0 to 21 d	690	697	690	698	689	2.680	0.984	0.526
22 to 42 d	1,373	1,439	1,418	1,458	1,366	20.200	0.976	0.072
0 to 42 d	2,063	2,136	2,108	2,155	2,055	20.300	0.979	0.055
FI^5^, g/bird								
0 to 21 d	968	969	985	979	971	3.520	0.550	0.234
22 to 42 d	2,533	2,559	2,536	2,562	2,539	10.300	0.761	0.408
0 to 42 d	3,501	3,527	3,521	3,541	3,510	10.100	0.614	0.242
FCR^6^								
0 to 21 d	1.40	1.39	1.43	1.40	1.41	0.005	0.593	0.672
22 to 42 d	1.85	1.78	1.79	1.76	1.87	0.023	0.863	0.059
0 to 42 d	1.70	1.65	1.67	1.64	1.71	0.014	0.799	0.058

^1^SPM, sprouted pearl millet.

^2^SEM, standard error of mean.

^3^Orthogonal polynomial (linear and quadratic) was used to examine the dose response due to increasing amounts of SPM in the diet. *P* ≤ 0.05 was considered to be statistically significant and *P* < 0.10 considered as trends.

^4^BWG, body weight gain.

^5^FI, feed intake.

^6^FCR, feed conversion ratio. *n* = 6.

The effect of SPM inclusion in the diets of broiler chickens on carcass traits is shown in Table 4. There was no effect on the carcass yield and the various carcass cut-up parts except drumstick and wings. The drumstick weight of broiler chickens showed quadratic effect (*P* = 0.044) while the wing weight showed linear effect (*P* = 0.047) on treatment diets at 21 d. The inclusion of SPM had no effect (*P* > 0.05) on carcass traits of broilers chickens at 42 d (Table 4).

**Table 4.** Carcass traits as percentage live weight of broiler chicken fed sprouted whole PM-based diets

Item	SPM^1^ (%)					SEM^2^	*P*-value^3^	
	0	25	50	75	100		Linear	Quadratic
0 to 21 d								
Carcass yield	57.00	56.40	56.80	57.20	56.60	0.310	0.981	0.983
Breast	22.50	22.00	21.60	22.50	25.10	0.540	0.195	0.156
Drumstick	9.08	9.28	9.60	9.00	8.58	0.130	0.135	0.044
Thigh	10.60	11.00	11.50	11.50	12.40	0.310	0.126	0.868
Wing	6.10	5.81	6.06	5.90	5.56	0.070	0.047	0.345
Abdominal fat	1.21	0.77	0.96	1.07	1.16	0.090	0.731	0.185
22 to 42 d								
Carcass yield	60.40	61.70	63.10	61.10	60.00	0.480	0.657	0.057
Breast	24.20	24.70	26.40	23.90	24.00	0.350	0.589	0.084
Drumstick	9.85	9.89	9.93	10.08	9.61	0.150	0.801	0.502
Thigh	12.20	12.50	12.60	12.60	12.40	0.190	0.658	0.531
Wing	5.98	6.33	6.18	6.13	5.82	0.079	0.385	0.110
Abdominal fat	1.17	1.22	0.93	0.92	1.16	0.097	0.720	0.531

^1^SPM, sprouted pearl millet.

^2^SEM, standard error of mean.

^3^Orthogonal polynomial (linear and quadratic) was used to examine the dose response due to increasing amounts of SPM in the diet. *P* ≤ 0.05 was considered to be statistically significant and *P* < 0.10 considered as trends. *n* = 6.

The organ weights of broiler chickens fed SPM-based diets are presented in Table 5. The various organ weights measured were not influenced by the experimental diets except the liver weights of broiler chickens, which showed linear response (*P* = 0.018) at 21 d and (*P* = 0.004) at 42 d to the treatment diets.

**Table 5.** Digestive and immune organ weights expressed as percentage live weight of broiler chickens fed sprouted whole PM-based diets

Item	SPM^1^ (%)					SEM^2^	*P*-value^3^	
	0	25	50	75	100		Linear	Quadratic
0 to 21 d								
Pancreas	0.31	0.31	0.32	0.30	0.28	0.006	0.146	0.412
Gizzard	2.13	1.99	2.04	1.97	1.99	0.033	0.226	0.487
Proventriculus	0.45	0.42	0.44	0.41	0.42	0.009	0.432	0.769
Liver	2.14	2.10	2.18	2.14	2.32	0.028	0.018	0.106
Bursa	0.19	0.19	0.18	0.21	0.18	0.008	0.470	0.933
Spleen	0.07	0.08	0.07	0.06	0.06	0.004	0.302	0.296
Thymus	0.40	0.34	0.47	0.44	0.42	0.022	0.318	0.704
22 to 42 d								
Pancreas	0.19	0.18	0.17	0.17	0.17	0.045	0.097	0.615
Gizzard	1.58	1.52	1.46	1.42	1.54	0.026	0.348	0.102
Proventriculus	0.26	0.27	0.27	0.24	0.25	0.073	0.259	0.619
Liver	1.38	1.50	1.48	1.47	1.74	0.035	0.004	0.235
Bursa	0.09	0.09	0.11	0.09	0.09	0.007	0.922	0.673
Spleen	0.06	0.06	0.06	0.05	0.06	0.039	0.613	0.925
Thymus	0.22	0.30	0.28	0.25	0.30	0.014	0.320	0.671

^1^SPM, sprouted pearl millet.

^2^SEM, standard error of mean.

^3^Orthogonal polynomial (linear and quadratic) was used to examine the dose response due to increasing amounts of SPM in the diet. *P* ≤ 0.05 was considered to be statistically significant and *P* < 0.10 considered as trends. *n* = 6.

The effect of SPM on blood hematology and biochemistry is presented in Table 6 and Table S1, respectively. The various hematological indices were not significant except MCHC, which showed quadratic response (*P* = 0.036) at 21 d to treatment diets. None of the serum biochemical indices tested were influenced except LDL concentration of broiler chickens, which presented linear increase (*P* = 0.026; Table 6 and Table S1) at 21 d.

**Table 6.** Biochemical and hematological profile of broiler chickens fed sprouted whole PM-based diets at 21 d

Item	SPM^1^ (%)					SEM^2^	*P*-value^3^	
	0	25	50	75	100		Linear	Quadratic
Cholesterol, mg/dL	105.00	108.00	118.00	113.00	123.00	7.450	0.160	0.718
HDL^4^, mg/dL	52.40	54.30	51.00	49.40	52.30	2.950	0.426	0.847
LDL^5^, mg/dL	37.20	39.00	41.50	45.00	48.00	4.020	0.026	0.813
Total protein, g/dL	2.78	2.99	2.94	2.63	2.69	0.067	0.240	0.319
Triglycerides, mg/dL	62.70	64.70	64.20	69.80	58.80	3.120	0.767	0.335
Lymphocytes, %	64.30	66.00	64.30	64.50	63.50	1.010	0.634	0.656
MCHC^6^, g/dL	32.90	32.60	32.50	32.70	33.60	0.160	0.133	0.036
MCV^7^, fL	95.90	91.00	91.30	89.50	95.00	4.360	0.917	0.603
Monocytes, %	2.83	3.33	2.67	3.17	2.50	0.160	0.483	0.408
PCV^8^, %	29.00	30.50	29.50	28.50	28.20	0.610	0.397	0.473
RBC^9^, × 10^6^/µL	3.08	3.14	3.21	3.11	3.11	0.049	0.892	0.470
WBC^10^, × 10^3^/µL	15,283	16,850	15,925	15,667	15,592	295	0.802	0.333

^1^SPM, sprouted pearl millet.

^2^SEM, standard error of mean.

^3^Orthogonal polynomial (linear and quadratic) was used to examine the dose response due to increasing amounts of SPM in the diet. *P* ≤ 0.05 was considered to be statistically significant and *P* < 0.10 considered as trends.

^4^HDL, high-density lipoprotein.

^5^LDL, low-density lipoprotein.

^6^MCHC, mean corpuscular hemoglobin concentration.

^7^MCV, mean corpuscular volume.

^8^PCV, packed cell volume.

^9^RBC, red blood cell.

^10^WBC, white blood cell; *n* = 6.

The relative length and weight of small intestinal segments and ceca in broiler chickens were measured (Table 7). The duodenal length showed linear decrease (*P* = 0.046) while quadratic effect (*P* = 0.032) was observed on ilea relative length of broiler chickens at 21 d. Jejunal and ileal length at 42 d both showed linear effect (*P* < 0.001) and (*P* = 0.016) on treatment diets. The jejunal weight of broiler chickens presented quadratic response (*P* = 0.034) at 21 d while no effect was observed at 42 d (Table 7).

**Table 7.** Relative length (cm/g) and relative weights (g/g) of small intestine in broiler chickens fed sprouted whole PM-based diets

Item	SPM^1^(%)					SEM^2^	*P*-value^3^	
	0	25	50	75	100		Linear	Quadratic
0 to 21 d								
*Relative length*								
Duodenum	2.92	2.97	2.71	2.79	2.75	0.041	0.046	0.576
Jejunum	5.77	6.10	6.26	5.80	5.88	0.110	0.788	0.148
Ileum	6.44	6.97	6.57	6.60	6.15	0.110	0.096	0.032
Caecum	1.53	1.63	1.40	1.55	1.53	0.062	0.758	0.877
*Relative weights*								
Duodenum	0.56	0.50	0.49	0.47	0.45	0.020	0.085	0.455
Jejunum	0.87	0.81	0.78	0.74	0.88	0.028	0.938	0.034
Ileum	0.71	0.86	0.73	0.73	0.62	0.039	0.219	0.224
Caecum	0.38	0.28	0.25	0.28	0.25	0.025	0.163	0.364
22 to 42 d								
*Relative length*								
Duodenum	1.36	1.37	1.28	1.36	1.19	0.031	0.075	0.378
Jejunum	3.31	3.07	2.83	2.94	2.72	0.059	<.001	0.360
Ileum	3.43	3.21	3.21	3.20	2.97	0.062	0.016	0.939
Caecum	0.83	0.78	0.74	0.65	0.77	0.029	0.238	0.257
*Relative weights*								
Duodenum	0.27	0.26	0.29	0.28	0.31	0.008	0.115	0.581
Jejunum	0.52	0.47	0.47	0.47	0.48	0.010	0.352	0.178
Ileum	0.45	0.44	0.46	0.43	0.42	0.013	0.346	0.609
Caecum	0.23	0.22	0.22	0.22	0.22	0.007	0.526	0.830

^1^SPM, sprouted pearl millet.

^2^SEM, standard error of mean.

^3^Orthogonal polynomial (linear and quadratic) was used to examine the dose response due to increasing amounts of SPM in the diet. *P* ≤ 0.05 was considered to be statistically significant and *P* < 0.10 considered as trends; *n* = 6.

The intestinal digesta pH of broiler chickens fed SPM-based diets is shown in Table 8. There was no effect of SPM-based diets on digesta pH in the various intestinal organs at 21 d. The pH of digesta in the crop presented both linear effect (*P* = 0.015) and quadratic response (*P* = 0.037), while proventriculus showed quadratic effect (*P* = 0.009) at 42 d.

**Table 8.** Intestinal digesta pH of broiler chickens fed sprouted whole PM-based diets

Item	SPM^1^(%)					SEM^2^	*P*-value^3^	
	0	25	50	75	100		Linear	Quadratic
0 to 21 d								
Crop	4.54	4.42	4.49	4.34	4.84	0.081	0.223	0.583
Proventriculus	2.60	3.51	3.14	3.26	3.69	0.180	0.580	0.208
Gizzard	2.46	2.91	2.84	2.75	2.71	0.110	0.336	0.264
Duodenum	5.46	5.6	5.94	5.98	5.91	0.110	0.212	0.865
Jejunum	5.74	5.72	5.69	5.65	5.76	0.032	0.173	0.559
Ileum	6.41	6.10	6.41	6.26	5.99	0.130	0.896	0.576
Caecum	5.89	5.88	5.9	5.92	5.91	0.072	0.964	0.947
22 to 42 d								
Crop	5.16	4.41	4.49	4.35	4.33	0.099	0.015	0.037
Proventriculus	2.81	1.99	2.86	3.39	3.10	0.170	0.164	0.009
Gizzard	3.10	2.68	2.96	3.43	2.90	0.100	0.332	0.054
Duodenum	5.62	5.76	5.77	5.80	5.88	0.040	0.391	0.350
Jejunum	5.79	5.74	5.68	5.74	5.71	0.031	0.566	0.462
Ileum	6.69	6.13	6.27	6.39	5.76	0.140	0.944	0.179
Caecum	5.97	5.59	5.77	5.78	5.90	0.097	0.565	0.531

^1^SPM, sprouted pearl millet.

^2^SEM, standard error of mean.

^3^Orthogonal polynomial (linear and quadratic) was used to examine the dose response due to increasing amounts of SPM in the diet. *P* ≤ 0.05 was considered to be statistically significant and *P* < 0.10 considered as trends; *n* = 6.

The *Lactobacilli* count in ceca of broiler chickens showed decreasing linear response (*P* = 0.010). However, there were no significant effect of treatment diets on *Salmonella* count in the ceca (Table 9).

**Table 9.** Ceca bacteria count of broiler chicken fed sprouted whole PM-based diets at 21 d

Item	SPM^1^(%)					SEM^2^	*P*-value^3^	
	0	25	50	75	100		Linear	Quadratic
*Lactobacilli* (Log CFU/g)	4.98	4.98	4.84	4.87	4.91	0.055	0.010	0.853
*Salmonella* (Log CFU/g)	5.22	5.23	5.32	5.23	5.32	0.032	0.846	0.096

^1^SPM, sprouted pearl millet

^2^SEM, standard error of mean.

^3^Orthogonal polynomial (linear and quadratic) was used to examine the dose response due to increasing amounts of SPM in the diet. *P* ≤ 0.05 was considered to be statistically significant and *P* < 0.10 considered as trends; *n* = 6.

## DISCUSSION

This research investigated the effect of SPM on the growth performance, intestinal development, bacterial count in the ceca, and blood indices of broiler chickens. The present study showed that sprouted whole PM had no significant effect on growth performance of broiler chickens. However, the best growth performance was obtained on partial inclusion of sprouted PM. Partial inclusion of SPM was observed to improve BWG by 4% to 5%, and lowered FCR by 4% at 42 d and 0 to 42 d. Similar findings were reported by other researchers on effect of sprouted feed on growth performance (Fanimo and Akinola, 2006; Maidala et al., 2016; Younis et al., 2019). The research study on milled sprouted PM showed that the substitution of millet had no effect on BWG, FI, and FCR of broiler chickens (Afsharmanesh et al., 2016). Results from this study also agree with the report that sprouted mung bean supplementation improved significantly the growth performance of broiler chickens infected with *Eimeria* spp. oocyst (Singh et al., 2013). The dietary inclusion of sprouted pulses reduced FCR of broiler chickens at day 21 while at day 42 no effect was observed on BWG and FCR (Rama Rao et al., 2018). Reports showed that inclusion of sprouted crushed barley in diet of parent flock hens improved growth and development in poultry accompanied by lowered feed conversion rate (Gadiev et al., 2021). Our results suggest that partial replacement of maize with whole SPM up to 75% improves their growth performance of broiler chickens.

The authors of this study could not find research in literature on the effect of sprouted PM on carcass characteristics of broiler chickens. However, the lack of treatment effect on carcass traits is similar to the earlier findings that reported that partial or complete replacement of yellow maize in diets of growing Japanese quail had no effect on carcass characteristics (Younis et al., 2019). The supplementation of sprouted pulses did not affect carcass variables (relative weights of breast, ready-to-cook yield, liver, and abdominal fat) in broiler chickens (Rama Rao et al., 2018). Research report showed that broiler chickens fed malted sorghum-based diets had similar carcass characteristics and meat quality traits compared to the control (Moses et al., 2022). Sprouted papaya seed meal significantly decreased breast meat yield in broiler chickens (Sugiharto et al., 2019). Results from this study are at variance with the reports that weaned rabbits fed sprouted sorghum had improved dressed weights compared to unsprouted sorghum (Aderemi and Wuraola, 2010).

The growth of broiler chickens in this study was not accompanied by significant effect of treatment diets on digestive and immune organ weights except for the relative liver weight. These findings are similar to earlier report in weaned rabbits that sprouted barley has no effect on internal organ characteristics except spleen and liver (Mohsen et al., 2015). The immunological organs of broilers were not affected by replacement of soybean with germinated and nongerminated *Mucuna pruriens* grain meal (Martínez-Pérez et al., 2016). However, in weaned rabbits, sprouted sorghum significantly lowered liver weight compared to the unsprouted and control diets (Aderemi and Wuraola, 2010). The liver is an organ involved in detoxification, nutrient metabolism, and maintaining cholesterol homeostasis (Kruit et al., 2006; Zaefarian *et al*., 2019). Increased relative liver size is considered as a positive indicator associated with higher metabolic activity (Zaefarian *et al*., 2019). The increased liver weight may be associated with increased LDL cholesterol concentration in broiler chickens fed sprouted whole PM.

The increased values for LDL cholesterol on sprouted whole PM indicated possible influence of treatment diets on nutrient utilization by broiler chickens. Younis et al. (2019) reported that germinated sorghum decreases blood serum total cholesterol and triglycerides compared to yellow corn in broiler chickens. LDL cholesterol is a low-density lipoprotein cholesterol that functions primarily as carrier of cholesterol from liver to the tissues (Superko et al., 2002). The possible explanation for high LDL cholesterol concentration on sprouted whole PM may be related to the fiber content. Previous research has reported a significant reduction of total and LDL cholesterol by small magnitude (Brown et al., 1999) in response to fiber level. The process of sprouting has been reported to breakdown complex compounds into simpler forms for efficient nutrient utilization (Oghbaei and Prakash, 2016).

Intestinal development is critical to achieve optimum growth performance and is reflected by the relative length and weight of organs (Wang et al., 2020; Alyileili et al., 2020). The longer the length of the intestine the greater will be the absorption of nutrients. The findings in this study showed that feeding sprouted whole PM has no stimulatory effect on the development of intestine in broiler chickens. Various factors have been reported to enhance intestinal development. The inclusion of whole sorghum has no effect on relative length of small intestine, ceca, and colon (Silva et al., 2015). Previous research showed that high dietary fiber contents increased length and weight of intestine of broiler chickens (Jørgensen et al., 1996; Alyileili et al., 2020). The effect of germination on dietary fiber content of sprouted grains is often inconsistent and depends on fiber fraction, germination time, and genotypes (Nelson et al., 2013). In germinated wheat, total dietary fibers decreased within the first 48 h of germination time (Koehler et al., 2007; Hung et al., 2012). In the current study, sprouting process was completed within 24 h and resulted in 25% decrease in fiber content of millet grain, which could affect intestinal development in broiler chickens.

The intestinal pH is involved in the regulation of microflora establishment within the intestinal tract. Beneficial microorganisms require acidic condition to grow and compete with pathogens while pathogens growth takes place at a pH close to 7 or higher. Low gizzard pH enhances mineral salts solubility and absorption as well as pepsin activity (Guinotte et al., 1995; Jiménez-Moreno et al., 2010). The proventriculus contains acidic secretions such as proteases (Svihus, 2011), which at low pH cause a decrease in the bacterial population (Yadav and Jha, 2019). The crop is a major site of bacterial activity (Yadav and Jha, 2019). It plays a major role in feed storage and moistening, as well as functional barrier for pathogens through decreasing pH value by microbial fermentation. The crop allows for a thorough utilization of exogenous enzymes by decreasing the value of digesta through *Lactobacilli* fermentation. In this study, SPM in diets corresponds to significant reduction of crop pH in contrast to the *Lactobacilli* count, which remained the same across diets.

Within the ceca, the present study revealed that sprouted whole PM influenced *Lactobacillus* which may be associated with improved growth performance (Blajman et al., 2017). It has been established that *Lactobacillus* are important in defense against infectious agents through competition with pathogenic bacteria in the intestine for adhesion sites and immune modulation (Liévin-Le Moal and Servin, 2014; Wang et al., 2017). Sorghum grain increased *Lactobacillus* population in small intestine and ceca with reduction in population of Clostridium in broiler chickens (Fagundes et al., 2017). Sprouted papaya seed meal improved lactic acid population while population of coliforms were reduced in broilers (Sugiharto et al., 2022).

## CONCLUSION

In conclusion, the results of this study showed that SPM has no adverse effect on growth performance of broiler chickens. The treatment diets increased LDL concentration. This study demonstrated contribution of sprouted whole pearl millet to low digesta pH in proventriculus. The length and weight of small intestine and ceca were altered while population of *Lactobacilli* enhanced in ceca by treatment diets. This study specifically demonstrated that sprouted whole pearl millet possesses the potential to improve BWG, FCR, and carcass traits without negative impact on organ developments, physiological status, *Lactobacilli* count, and health of broiler chickens.

## Supplementary Material

txad045_suppl_Supplementary_Table_S1
