# Supplementary material for: Effect of sprouted whole pearl millet on growth performance, intestinal development, bacterial count, and blood indices of broiler chickens
Source: Transl Anim Sci. 2023 May 8;7(1):txad045. doi: 10.1093/tas/txad045 (PMC10199787; doi:10.1093/tas/txad045)
Supplement: txad045_suppl_Supplementary_Table_S1 [file txad045_suppl_supplementary_table_s1.docx]

**Supplementary Table 1:** Hematological and biochemical profile of broiler chickens fed sprouted whole PM-based diet at 21 d.

| Item | SPM^1^ (%) | | | | | SEM^2^ | P-value^3^ | |
| --- | --- | --- | --- | --- | --- | --- | --- | --- |
|  | 0 | 25 | 50 | 75 | 100 |  | Linear | Quadratic |
| Basophils (%) | 1.50 | 2.17 | 2.50 | 1.67 | 2.17 | 0.370 | 0.586 | 0.410 |
| Hemoglobin (g/dl) | 9.53 | 9.93 | 9.67 | 9.43 | 9.27 | 0.190 | 0.453 | 0.499 |
| Heterophil (%) | 29.00 | 26.50 | 29.00 | 28.80 | 29.00 | 0.960 | 0.708 | 0.717 |
| MCH^4^ (pg) | 30.10 | 31.00 | 30.60 | 30.40 | 30.70 | 0.370 | 0.839 | 0.707 |
| Basophils (%) | 1.50 | 2.17 | 2.50 | 1.67 | 2.17 | 0.370 | 0.586 | 0.410 |
| Albumin (g/dl) | 1.46 | 1.54 | 1.58 | 1.46 | 1.49 | 0.047 | 0.684 | 0.233 |
| ALP^5^ (Unit/L) | 160.00 | 163.00 | 163.00 | 161.00 | 160.00 | 5.020 | 0.221 | 0.115 |
| ALT^6^ (Unit/L) | 20.70 | 20.00 | 21.00 | 20.20 | 17.80 | 1.340 | 0.507 | 0.596 |
| AST^7^ (Unit/L) | 104.60 | 96.10 | 102.00 | 93.10 | 90.20 | 3.220 | 0.160 | 0.884 |
| Creatinine (mg/dl) | 0.43 | 0.43 | 0.38 | 0.45 | 0.58 | 0.043 | 0.160 | 0.163 |
| Globulin (g/dl) | 1.32 | 1.45 | 1.36 | 1.17 | 1.20 | 0.067 | 0.330 | 0.654 |
| Glucose (mg/dl) | 208.00 | 214.00 | 226.00 | 215.00 | 208.00 | 3.900 | 0.740 | 0.128 |
| UREA (mg/dl) | 4.21 | 4.22 | 4.34 | 4.37 | 4.51 | 0.110 | 0.298 | 0.914 |

^1^SPM, sprouted pearl millet. ^2^SEM, standard error of mean; ^3^Orthogonal polynomial (linear and quadratic) were used to examine the dose response due to increasing amounts of SPM in the diet. P ≤ 0.05 was considered to be statistically significant and P < 0.10 as trends; ^4^MCH, Mean corpuscular hemoglobin; ^5^ALP, Alanine Phosphatase; ^6^ALT, Alanine Transaminase; ^7^AST, Aspartate Transaminase; n = 6;
